# Protective Effect of Lipoic Acid on Oxidative Stress and Tissue Damage Induced by Aflatoxin B_1_ in Young Laying Hens

**DOI:** 10.3390/toxins17040184

**Published:** 2025-04-06

**Authors:** Yihong Chu, Huanbin Wang, Xinyu Xu, Yun Ji, Yiting Zhao, Qianqian Yu, Shahid Ali Rajput, Yi Xue, Desheng Qi

**Affiliations:** 1 Department of Animal Nutrition and Feed Science, College of Animal Science and Technology, Huazhong Agricultural University, Wuhan 430070, China; cyhong@webmail.hzau.edu.cn (Y.C.); whbin@webmail.hzau.edu.cn (H.W.); xuxinyu7948@webmail.hzau.edu.cn (X.X.); jiy@webmail.hzau.edu.cn (Y.J.); yiting@webmail.hzau.edu.cn (Y.Z.); yuqianqian@webmail.hzau.edu.cn (Q.Y.); 2 Faculty of Veterinary and Animal Science, Muhammad Nawaz Shareef University of Agriculture, Multan 60000, Pakistan; shahid.ali@mnsuam.edu.pk; 3 China Grain Reserves (Sichuan) Quality Inspection Center Co., Ltd., Chengdu 610052, China; xueyi2025@yeah.net

**Keywords:** AFB_1_, lipoic acid, young laying hens, serum biochemistry, oxidative stress, tissue damage, This study investigated the protective effects of lipoic acid on oxidative stress and the liver, spleen, and ovarian tissue damage induced by aflatoxin B1 in young laying hens.

## Abstract

The aim of this study is to investigate the alleviating effect of lipoic acid on oxidative stress and tissue damage induced by aflatoxin B_1_ (AFB_1_) in young laying hens. The experiment was divided into a control group, an AFB_1_ group, and three lipoic acid treatment groups. The AFB_1_ group and three lipoic acid treatment groups were given diets supplemented with 90 μg/kg of AFB_1_. The additional amounts of lipoic acid were 20, 100, and 500 mg/kg, respectively, with a feeding period of 4 weeks. The experimental results showed that AFB_1_ significantly increased the levels of alanine aminotransferase (ALT), aspartate aminotransferase (AST), alkaline phosphatase (ALP) and malondialdehyde (MDA) in the serum and significantly decreased the levels of total protein (TP), albumin (ALB), total superoxide dismutase (T-SOD), glutathione peroxidase (GSH-Px) and catalase (CAT) (*p* < 0.05). In addition, AFB_1_ damaged the structure of the liver, spleen, and ovarian tissues. Lipoic acid reduced the levels of ALT, AST, ALP, and MDA in the serum and increased the levels of TP, ALB, T-SOD, GSH-Px, and CAT (*p* < 0.05). Meanwhile, lipoic acid also protected mitochondrial structure and alleviated liver, spleen, and ovarian tissue damage caused by AFB_1_. In summary, lipoic acid can alleviate oxidative stress and tissue damage caused by AFB_1_ in young laying hens.

## 1. Introduction

Aflatoxin B_1_ (AFB_1_) is a very common fungal toxin in feed, with high toxicity and a wide contamination range, posing a serious threat to the health of livestock and poultry [1,2]. Under the regulatory provisions of China’s Feed Hygiene Standard GB 13078-2017, the maximum permissible level of AFB_1_ in feed raw materials is strictly limited to 50 µg/kg. As typical feed-dependent economic animals, laying hens are prone to health damage through long-term intake of AFB_1_-contaminated feed [3]. Youth is a critical stage in the growth and development of laying hens and determines their later egg-laying performance [4]. Therefore, the developmental status of young laying hens will directly affect the economic benefits of breeding [4]. AFB_1_ in feed can harm the health of young laying hens [1,2]. After ingestion, AFB_1_ can cause damage to multiple systems in young laying hens, ranging from impaired growth performance, such as slow weight gain and a reduced feed conversion rate, to oxidative damage and damage to important organs such as the liver and spleen, leading to immune suppression [5,6]. At the same time, it increases the susceptibility of the chicken flock to various diseases, seriously affecting the growth and development of young laying hens and their overall health [7]. After AFB_1_ enters the cell, it leads to the production of a large amount of reactive oxygen species to attack unsaturated fatty acids on the mitochondrial membrane, causing lipid peroxidation, disrupting the integrity of the mitochondrial membrane structure, and further leading to mitochondrial dysfunction and cell apoptosis [8,9].

Lipoic acid is a naturally occurring dithiol compound, which is similar to the vitamin B class of compounds [10]. It is a strong antioxidant, usually in the form of lipoic acid, and its antioxidant capacity is stronger than that of common antioxidants such as vitamin C and vitamin E [11]. In the body, lipoic acid is synthesized by the lipoic acid synthase system in mitochondria [12]. It can directly eliminate reactive oxygen species in the body through oxidation-reduction or convert reactive oxygen species into less aggressive products, reducing the damage of reactive oxygen species to mitochondria [13]. When laying hens suffer from AFB_1_ poisoning, mitochondrial damage can also lead to a decrease in the synthesis ability of lipoic acid and an increase in the production of reactive oxygen species [9,14]. Therefore, it is reasonable to speculate that supplementation with lipoic acid in feed can alleviate or eliminate the harm of AFB_1_ to young laying hens.

To date, there have been no reports on the use of lipoic acid to alleviate AFB_1_ poisoning in young laying hens. Considering the significant harm done by AFB_1_ to laying hens [3,6], this study used young laying hens as experimental animals. The effects of different treatments on the production performance, organ weight, antioxidant enzyme content, serum biochemical indicators, liver, spleen, and ovarian tissue structure damage, and nuclear and mitochondrial structure damage in the livers of young laying hens were determined. The effects of AFB_1_ on oxidative stress and tissue damage in young laying hens, as well as the alleviating effect of lipoic acid on AFB_1_ poisoning symptoms, were explored. This study can provide a theoretical basis for the prevention of AFB_1_ poisoning in young laying hens during the breeding production process.

## 2. Results

### 2.1. Changes in Production Performance and Organ Weight

The initial weight of each group of young laying hens was similar, about 1.23 kg/hen. Adding 90 μg/kg AFB_1_ to the diet had no significant effect on the weight, daily feed intake, and heart and spleen weight of young laying hens (*p* > 0.05) (Table 1). The liver weight of the AFB_1_ group showed an increasing trend (*p* > 0.05) (Table 1). Compared with the AFB_1_ group, adding 100 mg/kg of lipoic acid to the diet during the fourth week significantly reduced liver weight (*p* < 0.05) (Table 1). When 20 mg/kg or 500 mg/kg of lipoic acid was added to the diet, there was a decreasing trend in liver weight (*p* > 0.05) (Table 1).

### 2.2. Changes in Serum Biochemical Indicators

AFB_1_ significantly increased the levels of ALT, AST, and ALP in the serum of young laying hens while significantly reducing the levels of TP and ALB in the serum (*p* < 0.05) (Table 2). Compared with the AFB_1_ group, when 100 or 500 mg/kg of lipoic acid was added to the diet, the levels of ALT, AST, and ALP in the serum of young laying hens significantly decreased, while the levels of TP and ALB significantly increased (*p* < 0.05) (Table 2). Compared with the control group, the serum total bile acid (TBA) content in the AFB_1_ group showed an upward trend (*p* > 0.05) (Table 2).

### 2.3. Changes in Serum Antioxidant Enzyme Levels

AFB_1_ significantly reduced the levels of T-SOD, GSH-Px, and CAT in serum and significantly increased the level of MDA (*p* < 0.05) (Table 3). Compared with the AFB_1_ group, adding 20 mg/kg of lipoic acid to the diet had no significant effect on the content of serum antioxidant enzymes (*p* > 0.05) (Table 3). When the dose of lipoic acid was 100 mg/kg or above, the levels of T-SOD and GSH-Px in serum significantly increased, while the level of MDA significantly decreased (*p* < 0.05) (Table 3). When the dose of lipoic acid was 500 mg/kg, the content of CAT in serum significantly increased (*p* < 0.05) (Table 3).

### 2.4. Changes in Liver Tissue Structure

**Figure 1:**
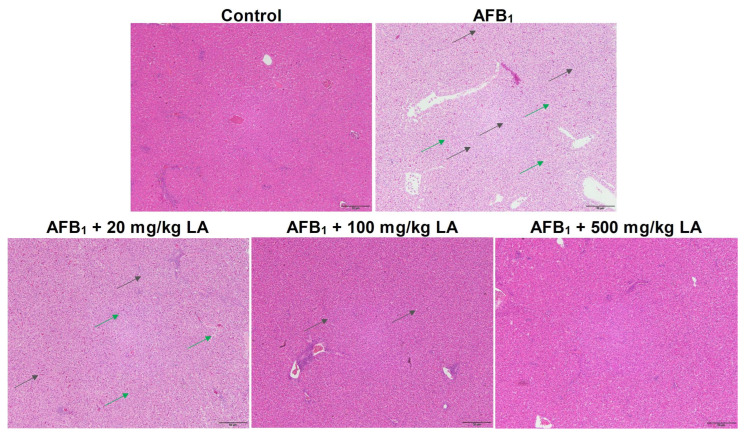
Changes in liver tissue structure of young laying hens fed by AFB_1_ and lipoic acid (LA) after the 4 week treatment. Green arrow: increased tissue spaces; black arrow: circular vacuoles formed by fat degeneration; the magnification is 100×.

The hierarchy of liver tissue structure in the control group was clear, with liver cells of regular morphology arranged close to each other and no symptoms such as fat degeneration or increased tissue gaps observed (Figure 1). There were symptoms of increased interstitial space and fatty degeneration in the liver tissue of the AFB_1_ group, and there were many circular vacuoles formed due to fatty degeneration on the slices (Figure 1). Adding 20 mg/kg of lipoic acid to the diet had a poor relief effect on liver AFB_1_ poisoning symptoms (Figure 1). When the amount of added lipoic acid reached 100 mg/kg, the symptoms of enlarged liver tissue gaps disappeared, and when the amount of added lipoic acid reached 500 mg/kg, the symptoms of hepatic steatosis disappeared (Figure 1).

### 2.5. The Condition of Hepatic Steatosis

**Figure 2:**
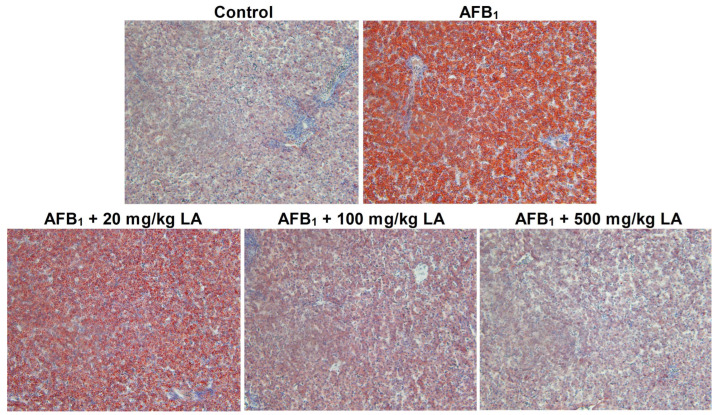
Changes in liver fatty degeneration of young laying hens fed by AFB_1_ and lipoic acid (LA) after the 4 week treatment. Red indicates the condition of fat degeneration; the magnification is 200×.

The livers of the control group were normal without any fat degeneration (Figure 2). There were obvious signs of steatosis in the livers of the AFB_1_ group, and a large amount of lipids were stained orange-red in the field of view (Figure 2). Compared with the AFB_1_ group, adding lipoic acid to the diet can alleviate the fat degeneration caused by AFB_1_. When the amount of lipoic acid added reached 500 mg/kg, the symptoms of fat degeneration disappeared (Figure 2).

### 2.6. Changes in Liver Ultrastructure

**Figure 3:**
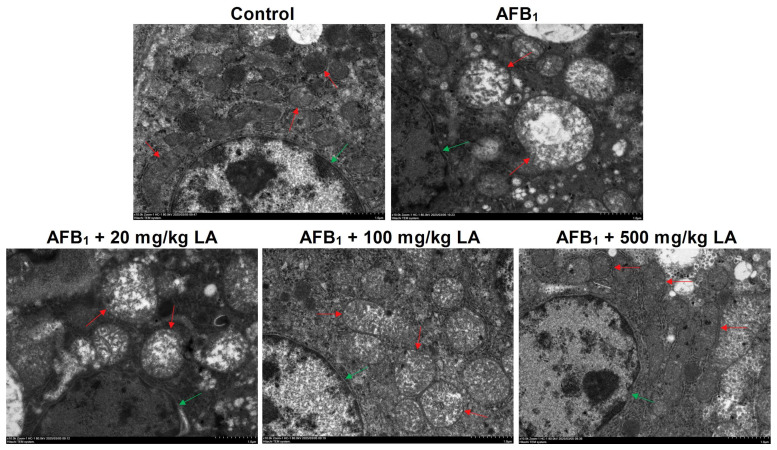
Changes in nuclear and mitochondrial structures in the liver of young laying hens fed by AFB_1_ and lipoic acid (LA) after the 4 week treatment. Red arrow: mitochondrion (AFB_1_ causes mitochondria to swell and mitochondrial cristae to break); green arrow: nucleus (AFB_1_ causes nuclear membrane shrinking); the magnification is 10,000×.

The contour of the nucleus and mitochondria in the control group liver is clear, the nuclear membrane of the nucleus is intact, the mitochondrial matrix is full, and the color of the matrix is darker (Figure 3). The nuclear membrane of AFB_1_ group liver cells shrinks, the mitochondria swell and enlarge, mitochondrial cristae break, and the matrix decreases and becomes partially blank (Figure 3). Adding lipoic acid to the diet can alleviate the nuclear and mitochondrial structural damage caused by AFB_1_. When the dose of lipoic acid is 100 mg/kg, the symptoms of nuclear membrane wrinkling disappear, and the symptoms of mitochondrial swelling and mitochondrial cristae damage are relieved (Figure 3). When the additional amount of lipoic acid is 500 mg/kg, the structure of the nucleus and mitochondria returns to normal (Figure 3).

### 2.7. Changes in the Structure of Spleen Tissue

**Figure 4:**
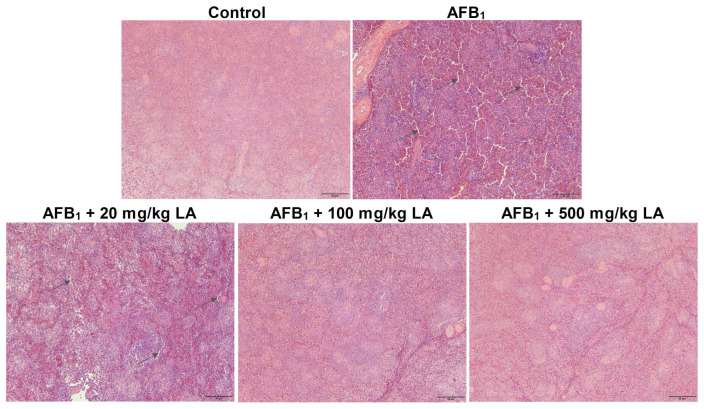
Changes in the spleen tissue structure of young laying hens fed by AFB_1_ and lipoic acid (LA) after the 4 week treatment. Black arrow: increased tissue spaces (organizational damage); the magnification is 100×.

The structural hierarchy of white and red pulp in the spleen of the control group of young laying hens is clear, and the structure of cells and tissues is normal (Figure 4). There was a symptom of increased tissue space in the spleen of the AFB_1_ group (Figure 4). Adding lipoic acid to the diet can alleviate spleen damage caused by AFB_1_. When the amount of lipoic acid added reaches 100 mg/kg or 500 mg/kg, the symptoms of enlarged spleen tissue gaps caused by AFB_1_ disappear (Figure 4).

### 2.8. Changes in Ovarian Tissue Structure

**Figure 5:**
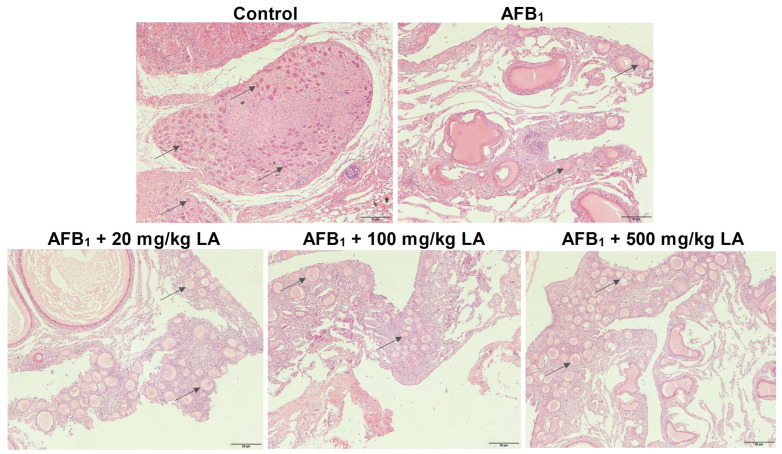
Changes in ovarian tissue structure of young laying hens fed by AFB_1_ and lipoic acid (LA) after the 4 week treatment. Black arrow: ovarian follicle (AFB_1_ reduces the number of ovarian follicles); the magnification is 100×.

The tissue structure of the control group’s ovaries is normal, and many follicles can be seen in the ovarian cortex (Figure 5). Compared with the control group, the number of follicles in the ovarian cortex of the AFB_1_ group decreased significantly, indicating that AFB_1_ caused damage to ovarian tissue (Figure 5). As the amount of added lipoic acid gradually increased, there was a significant increase in the number of follicles in the ovarian cortex, indicating that lipoic acid can alleviate ovarian tissue damage caused by AFB_1_ (Figure 5).

## 3. Discussion

The production performance and health status of laying hens are closely related to the breeding efficiency. Numerous studies have shown that AFB_1_ can adversely affect animal production performance [3,6,9,15,16]. Karimi Torshizi and Sedaghat (2023) reported that adding 500 μg/kg AFB_1_ to the diet significantly reduced the feed intake, egg production rate, and egg weight of laying hens and significantly increased the feed conversion rate [3]. Seifi et al. (2022) reported that adding 1000 μg/kg AFB_1_ to the diet significantly reduced the egg production rate and eggshell thickness of laying hens [17]. Yu et al. (2024) reported that adding 90 μg/kg AFB_1_ to the diet can have adverse effects on the production performance and organ health of meat ducks [15]. However, this study found that adding 90 μg/kg AFB_1_ to the diet had no significant effect on the production performance and organ weight of young laying hens, which may be due to their relative insensitivity to AFB_1_ [18]. Ducks are more sensitive to AFB_1_, while chickens are relatively insensitive to AFB_1_ [18]. Therefore, adding 90 μg/kg of AFB_1_ to the diet may have strong toxicity to meat ducks, but it may only be a low-dose toxicity to laying hens. In previous reports, the production performance of laying hens was only affected when the addition of AFB_1_ in the diet reached 500 or 1000 μg/kg [3,17]. Meanwhile, Raj et al. (2023) reported that adding 50 μg/kg AFB_1_ to the diet had no significant effect on the production performance of laying hens aged 246–259 days [19]. The results of this study are similar to the report, which further indicates that young laying hens have a high tolerance to AFB_1_.

Serum biochemical indicators can reflect the health status of the liver, and AFB_1_ can damage the health of animal livers [9,20,21]. This study found that the levels of ALT, AST, and ALP in the serum of young laying hens in the AFB_1_ group increased, while the levels of TP and ALB decreased, indicating that AFB_1_ also caused damage to the livers of young laying hens. This study also found that adding lipoic acid to the diet can alleviate the abnormal serum biochemical indicators caused by AFB_1_. Li et al. (2014) reported that adding 300 mg/kg of lipoic acid to the diet can alleviate the inflammation caused by AFB_1_ in broiler chickens, while increasing the levels of TP and ALB in the serum [22]. The results of this study are similar to the report, indicating that lipoic acid can alleviate liver damage in young laying hens caused by AFB_1_.

Antioxidant indicators can reflect the body’s ability to resist oxidative stress [23]. Numerous studies have shown that AFB_1_ can cause damage to the liver’s antioxidant system [24,25,26]. This study found that AFB_1_ reduces the levels of antioxidant enzymes T-SOD, GSH-Px, and CAT in serum, while increasing the level of lipid peroxidation product MDA, indicating that AFB_1_ caused oxidative stress in young laying hens. This study also found that adding lipoic acid to the diet can increase the content of antioxidant enzymes in serum and reduce the content of MDA. Maciejczyk et al. (2022) reported that lipoic acid can enhance the antioxidant capacity in the hypothalamus of rats, inhibiting oxidative stress and inflammation [27]. Skibska et al. (2023) reported that lipoic acid can increase the levels of GSH, SOD, and free thiol groups in rat kidneys, reduce the level of hydrogen peroxide and alleviate lipopolysaccharide induced oxidative stress [28]. Yang et al. (2023) reported that adding lipoic acid to the diet can increase the levels of SOD, CAT, and GSH Px in sheep serum, enhancing their antioxidant capacity [29]. The results of this study are similar to those reported above, indicating that lipoic acid can enhance the antioxidant capacity of young laying hens and alleviate oxidative damage caused by AFB_1_.

The liver is a target organ of AFB_1_; therefore, when animals suffer from AFB_1_ poisoning, the damage to the liver is extremely severe [15,16,18]. This study found that AFB_1_ caused fat degeneration and increased tissue space in the liver of young laying hens, indicating that AFB_1_ also caused tissue damage to the liver of young laying hens. This result is consistent with previous reports. This study also found that lipoic acid can alleviate liver damage in young laying hens caused by AFB_1_. Li et al. (2014) reported that adding 300 mg/kg of lipoic acid to the diet can alleviate AFB_1_-induced liver damage in broiler chickens [22]. Longhitano et al. (2024) reported that lipoic acid can increase the antioxidant level of liver cells and alleviate oxidative stress damage in liver cells [30]. The results of this study are consistent with previous reports. The important reason for the relief of liver damage symptoms may be related to the increased antioxidant capacity of young laying hens by lipoic acid [13,31].

AFB_1_ can disrupt the structure of the nucleus and mitochondria, causing cell apoptosis [9,16]. In this study, it was also found that AFB_1_ can cause structural damage to the nuclei and mitochondria in the livers of young laying hens. At the same time, this study also found that lipoic acid can relieve symptoms such as mitochondrial swelling, decreased matrix, and damaged mitochondrial cristae caused by AFB_1_. Longhitano et al. (2023) reported that lipoic acid can not only restore the damaged membrane potential of mitochondria in steatosis model cells but also improve the symptoms of mitochondrial cristae fragmentation [32]. Mozaffarian et al. (2022) reported that adding 40 μM or more of lipoic acid to the culture medium can alleviate mitochondrial membrane damage caused by arsenic [33]. In addition, Song et al. (2022) reported that adding 100 mg/kg of lipoic acid to the diet can alleviate mitochondrial structural damage in heart cells of chronic sleep-deprived (CSD) mice [34]. The results of this study are similar to those reported above, indicating that lipoic acid can relieve mitochondrial damage in the livers of young laying hens caused by AFB_1_. In young laying hens, lipoic acid is synthesized by mitochondria and can clear reactive oxygen species [12,35]. AFB_1_ causes damage to mitochondrial structure, which inevitably inhibits the synthesis process of lipoic acid [12,35]. By adding lipoic acid to the diet, the insufficient synthesis of lipoic acid in mitochondria is compensated for, and the antioxidant capacity of the liver is improved. Therefore, the damaged mitochondrial structure in the liver is relieved.

The spleen and ovaries are important immune and reproductive organs in laying hens, respectively. This study found that AFB_1_ caused damage to the tissue structure of the spleen and ovaries of young laying hens. Zhu et al. (2017) found that adding 600 μg/kg AFB_1_ to the diet can cause spleen congestion in one-day-old broiler chickens and increase the number of apoptotic cells in the spleen [36]. Wu et al. (2023) reported that adding 2000 μg/kg AFB_1_ to the diet increased the number of blocked follicles in the ovaries of rats and damaged ovarian health [37]. This study also found that adding lipoic acid to the diet can partially alleviate the structural damage of the spleen and ovarian tissues caused by AFB_1_. The reason may be that lipoic acid enhances the antioxidant capacity of spleen and ovarian tissues, or it may be that lipoic acid accelerates the metabolic process of AFB_1_ in the liver, reducing the toxic effects of AFB_1_ on the body [13,18,31]. Further research is needed to determine the specific causes. Damage to ovarian structure may affect the egg laying performance of laying hens in the later stage, so special attention should be paid to the prevention and control of AFB_1_ in laying hen feed during production.

## 4. Conclusions

In summary, adding 90 μg/kg AFB_1_ to the diet does not significantly affect the production performance of young laying hens, but AFB_1_ can damage the structure of mitochondria in the livers of laying hens and impair the health of liver, spleen, and ovarian tissues. Adding lipoic acid to the diet can enhance the antioxidant capacity of young laying hens, protect mitochondrial structure, and alleviate symptoms such as abnormal serum biochemical indicators, oxidative stress, and damage to the liver, spleen, and ovarian tissues caused by AFB_1_.

## 5. Materials and Methods

### 5.1. Experimental Design and Sample Collection Methods

This experiment was approved by the Ethics Committee of Huazhong Agricultural University with approval number HZAUCH-2025-0001. A total of 160 105-day-old Jingfen No. 6 young laying hens with similar appearance and weight were selected for the experiment. All young laying hens were randomly divided into five groups (n = 8 repetitions/group, 4 hens/repetitions). The AFB_1_ group and three lipoic acid treatment groups were all fed a basic diet supplemented with 90 μg/kg AFB_1_. The basic diets of the three lipoic acid treatment groups were also supplemented with 20, 100, and 500 mg/kg lipoic acid, respectively. The formula and nutritional levels of the basic diets are shown in Supplementary Table S1. All young laying hens were raised in egg cages at the animal experimental base of Huazhong Agricultural University. During the experiment, all young laying hens were free to feed and drink, and the feeding period was 4 weeks. During the feeding period, the feed intake and weights of the young laying hens were recorded. After the fourth week, one chicken was randomly selected from each replicate, and blood was collected through the Infrawinged vein. After serum precipitation, the serum was separated using a low-temperature high-speed centrifuge (Eppendorf AG, Hamburg, Germany) at 3000 r/min. The serum was then stored in a −80 °C freezer for the determination of serum biochemical and antioxidant indicators. After the blood collection was completed, the young laying hens were euthanized by the acute blood loss method, and the abdominal cavity of the hens was opened for sampling. Livers, spleens, and hearts were weighed, and the weights were recorded. Small samples of liver, spleen, and ovary tissues were collected for tissue section preparation.

### 5.2. Determination of Growth Performance

During the experiment, the feeding amount and remaining feed amount of each replicate were weighed and recorded, and the average daily feed intake was calculated based on the feeding amount and remaining feed amount. Livers, spleens, and hearts were weighed, and the weights were recorded. Small samples of liver, spleen, and ovary tissues were collected for tissue section preparation.

### 5.3. Determination of Organ Weight

During slaughtering and sampling, the intact liver, spleen, and heart were removed; the weights of the liver, spleen, and heart were accurately weighed, and the data were recorded.

### 5.4. Determination of Serum Biochemical Indicators

The levels of serum biochemical indicators were measured using a fully automated biochemical analyzer (Beckman, Brea, CA, USA) and the appropriate test kits (Shenzhen Mindray Biomedical Technology Co., Ltd., Nanjing, China), and the methods for detecting each indicator were the same as those described in the kit instructions. The serum was stored at −80 °C until use.

### 5.5. Determination of Serum Antioxidant Indicators

The levels of antioxidant indicators were detected using commercial reagent kits from Nanjing Jiancheng Biotechnology Co., Ltd. (Nanjing, China), and the methods for detecting each indicator were the same as those described in the kit instructions.

### 5.6. H&E Staining of the Liver, Spleen, and Ovary

Fresh liver, spleen, and ovarian tissue blocks were immersed in a 4% paraformaldehyde solution for 24 h, and then the tissue blocks were dehydrated in a dehydrator (Junjie Electronics Co., Ltd., Wuhan, China). The dehydrated tissue blocks were made into wax blocks using paraffin and then sliced into sections with a thickness of about 4 μm using a microtome (Leica Instruments Co., Ltd., Shanghai, China). After transferring the slices onto a glass slide, the cell nucleus was first stained with haematoxylin staining solution, followed by staining of the cytoplasm with eosin staining solution. After the staining was completed, the slices were dehydrated and air dried. The surface of the slice was covered with a cover glass and sealed with neutral gum. Histomorphological analysis was performed using a motorized bright-field microscope (BX53, Olympus, Tokyo, Japan) with integrated cellSens imaging software (version 1.12).

### 5.7. Liver Oil Red O Staining

After being dehydrated and embedded, the tissue blocks were sliced into sections with a thickness of about 8 μm using a cryomicrotome (Thermo, Waltham, MA, USA). After reheating and drying the slices, they were fixed with fixative and then stained with Oil Red O staining solution. After diluting the background with 75% alcohol, the cell nucleus was stained with haematoxylin staining solution. The surface of the slice was covered with a cover glass and sealed with neutral gum. Detection analysis was performed using a motorized bright-field microscope (BX53, Olympus, Tokyo, Japan) with integrated cellSens imaging software.

### 5.8. Observation of Liver Ultrastructure

Fresh liver tissue blocks were soaked in 2.5% glutaraldehyde solution for 24 h and washed three times with a phosphate-buffer solution. The cleaned tissue blocks were then fixed in osmium acid solution for 2 h and washed three times with the phosphate-buffer solution. After dehydrating the tissue block with alcohol, the epoxy resin was then allowed to infiltrate the tissue block. After embedding the tissue block with pure Spurr resin, the tissue block was sliced into ultra-thin sections of approximately 70 nm using an ultramicrotome (Leica Instruments Co., Ltd., Shanghai, China) with a diamond knife. The sections are floated on the water surface, picked up and fixed with copper grids, then stained with uranyl acetate and lead citrate, and finally air-dried at room temperature. Ultrastructural analysis was conducted at 80 kV accelerating voltage using a field-emission transmission electron microscope (Hitachi HT7800, Hitachi High-Tech, Tokyo, Japan) equipped with an AMT XR111 digital imaging system. Calibrated magnifications (×10,000) were applied for systematic observation of epoxy resin-embedded sections (70–90 nm thickness).

### 5.9. Statistical Analysis

After preliminary sorting of the experimental data using Excel software, SPSS Statistics 25 (IBM Corp. Armonk, NY, USA) was used for statistical analysis. Using one-way ANOVA and Tukey’s test to analyze between-group differences.

## Figures and Tables

**Table 1:** Changes in the production performance and organ weight of young laying hens fed by AFB_1_ and lipoic acid (LA) after the 4 week treatment ^1^.

Items	Control	AFB_1_	AFB_1_ + 20 mg/kg LA	AFB_1_ + 100 mg/kg LA	AFB_1_ + 500 mg/kg LA
Week 2					
Weight (kg)	1.42 ± 0.03	1.37 ± 0.04	1.41 ± 0.06	1.40 ± 0.09	1.39 ± 0.10
Daily feed intake (g)	93.80 ± 2.73 ^a^	89.82 ± 2.38 ^ab^	87.28 ± 6.96 ^b^	89.91 ± 3.19 ^ab^	88.77 ± 4.81 ^b^
Week 4					
Weight (kg)	1.49 ± 0.03	1.47 ± 0.06	1.49 ± 0.09	1.49 ± 0.09	1.44 ± 0.09
Daily feed intake (g)	94.00 ± 3.71	92.80 ± 4.76	90.03 ± 10.08	91.62 ± 3.27	89.24 ± 5.27
Heart (g)	6.28 ± 1.13	6.93 ± 1.41	6.34 ± 0.82	6.87 ± 1.35	6.35 ± 0.81
Liver (g)	34.43 ± 6.69 ^ab^	39.71 ± 8.45 ^a^	35.10 ± 5.47 ^ab^	32.03 ± 4.85 ^b^	33.12 ± 4.34 ^ab^
Spleen (g)	3.15 ± 0.59	3.06 ± 0.60	2.81 ± 0.47	3.26 ± 0.40	3.02 ± 0.43

^1^ Values are expressed as means ± SD (n = 8), and different lowercase letters indicate significant differences between groups (*p* < 0.05).

**Table 2:** Changes in serum biochemical indicators of young laying hens fed by AFB_1_ and lipoic acid (LA) after the 4 week treatment.

Bioindicators	Control	AFB_1_	AFB_1_ + 20 mg/kg LA	AFB_1_ + 100 mg/kg LA	AFB_1_ + 500 mg/kg LA
ALT (U/L)	1.43 ± 0.27 ^d^	3.36 ± 0.55 ^a^	3.39 ± 0.44 ^a^	2.61 ± 0.38 ^b^	1.96 ± 0.37 ^c^
AST (U/L)	195.78 ± 19.37 ^c^	293.73 ± 18.44 ^a^	281.29 ± 16.10 ^a^	250.90 ± 18.60 ^b^	211.11 ± 13.72 ^c^
ALP (U/L)	288.23 ± 47.06 ^c^	474.99 ± 40.13 ^a^	470.80 ± 45.79 ^a^	381.59 ± 30.33 ^b^	315.33 ± 61.57 ^c^
TP (g/L)	47.79 ± 6.17 ^a^	16.69 ± 3.70 ^d^	17.53 ± 3.98 ^d^	26.80 ± 5.51 ^c^	36.48 ± 4.31 ^b^
ALB (g/L)	22.44 ± 3.87 ^a^	10.35 ± 1.97 ^d^	11.18 ± 2.85 ^d^	14.24 ± 1.91 ^c^	17.71 ± 3.02 ^b^
TBA (μmol/L)	37.39 ± 4.93	41.01 ± 7.19	40.88 ± 7.21	39.20 ± 9.16	36.49 ± 5.85

Values are expressed as means ± SD (n = 8), and different lowercase letters indicate significant differences between groups (*p* < 0.05).

**Table 3:** Changes in serum antioxidant enzyme levels of young laying hens fed by AFB_1_ and lipoic acid (LA) after the 4 week treatment.

Antioxidant Enzymes	Control	AFB_1_	AFB_1_ + 20 mg/kg LA	AFB_1_ + 100 mg/kg LA	AFB_1_ + 500 mg/kg LA
T-SOD (U/mL)	89.02 ± 7.88 ^a^	60.06 ± 6.03 ^d^	60.18 ± 4.52 ^d^	67.48 ± 4.19 ^c^	77.64 ± 5.80 ^b^
MDA (nmol/mL)	7.80 ± 0.53 ^c^	16.36 ± 1.36 ^a^	15.78 ± 1.57 ^a^	12.69 ± 1.13 ^b^	8.80 ± 1.69 ^c^
GSH-Px (U/mL)	1862.84 ± 63.03 ^a^	1371.62 ± 137.74 ^c^	1397.30 ± 81.93 ^c^	1642.57 ± 126.95 ^b^	1775.68 ± 61.16 ^a^
CAT (U/mL)	3.34 ± 0.46 ^a^	1.82 ± 0.56 ^c^	1.91 ± 0.52 ^c^	2.25 ± 0.35 ^bc^	2.58 ± 0.33 ^b^

Values are expressed as means ± SD (n = 8), and different lowercase letters indicate significant differences between groups (*p* < 0.05).

## Data Availability

The data presented in this study are available on request from the corresponding author (the data are not publicly available due to privacy or ethical restrictions).

## Supplementary Materials

The following supporting information can be downloaded at: https://www.mdpi.com/article/10.3390/toxins17040184/s1, Table S1: Composition of basal diets and nutrient levels.
