# Supplementary material for: Protective Effect of Lipoic Acid on Oxidative Stress and Tissue Damage Induced by Aflatoxin B1 in Young Laying Hens
Source: Toxins (Basel). 2025 Apr 6;17(4):184. doi: 10.3390/toxins17040184 (PMC12031067; doi:10.3390/toxins17040184)
Supplement: Supplementary file 1 [file toxins-17-00184-s001.zip › toxins-3557687-supplementary.pdf]

Supplementary Table 1. Composition of basal diets and nutrient levels[1].

Ingredients	Percentage (%)	Nutrient level	Content
Corn	64.82	ME (kcal/kg)	2800
Soybean meal	19.30	Crude protein (%)	16.06
Wheat bran	10.00	Calcium (%)	0.91
Soybean oil	1.17	Available phosphorus (%)	0.48
Calcium monophosphate	2.00	Met (%)	0.44
Limestone	1.02	Lys (%)	0.84
Lys	0.09	Thr (%)	0.62
Met	0.17		
Thr	0.04		
Salt	0.30		
Choline chloride	0.09		
Premix	1.00		
Total	100		

[1]The premix provided the following per kilogram of diet: vitamin A, 12,500 IU; cholecalciferol 4125 IU; vitamin E, 25 mg; thiamine, 2.50 mg; riboflavin, 10 mg; pyridoxine, 6 mg; iron, 65 mg; manganese, 65 mg; copper, 15 mg; zinc, 66 mg; selenium, 0.4 mg; iodine, 1.0 mg.
